# Psychometric Properties of the Spanish Version of the Gender Ideology and LGBTQ+ Lobby Conspiracies Scale (GILC): Evidence from a Chilean Sample

**DOI:** 10.3390/bs16081375

**Published:** 2026-08-11

**Authors:** Carlos Calderón-Carvajal, Leandro Vergara-Toro, Siu Lay-Lisboa

**Affiliations:** School of Psychology, Universidad Católica del Norte, Antofagasta 127070, Chile; ccalderon@ucn.cl (C.C.-C.); slay@ucn.cl (S.L.-L.)

**Keywords:** conspiracy beliefs, LGBTQ+, scale adaptation, psychometric properties, confirmatory factor analysis, measurement invariance, validity evidence

## Abstract

Conspiracy beliefs targeting lesbian, gay, bisexual, transgender, and queer (LGBTQ+) individuals have attracted increasing attention in social and political psychology due to their role in prejudice, social exclusion, and resistance to social change. Despite growing interest in this phenomenon, validated instruments for assessing LGBTQ+-related conspiracy beliefs in Spanish-speaking populations remain limited. The present study aimed to adapt and evaluate the psychometric properties of the Spanish version of the Gender Ideology and LGBTQ+ Lobby Conspiracies Scale (GILC) in a Chilean sample. A total of 611 participants completed the instrument. Evidence based on internal structure was examined using parallel analysis, psychometric network analysis, confirmatory factor analysis (CFA), and measurement invariance testing across sex assigned at birth. Evidence based on relations with external variables was assessed through sociodemographic characteristics, religiosity, political orientation, and generic conspiracy beliefs. Results consistently supported a predominantly unidimensional structure. The CFA showed that the original one-factor model achieved satisfactory fit after incorporating two theoretically meaningful residual correlations. In addition, the scale demonstrated configural, metric, and scalar invariance across sex assigned at birth. GILC scores were positively associated with generic conspiracy beliefs, religiosity, age, and right-wing political orientation. Overall, the findings provide support for the validity of the Spanish version of the GILC and suggest that it constitutes a useful tool for studying LGBTQ+-related conspiracy beliefs in Spanish-speaking contexts.

## 1. Introduction

Conspiracy beliefs have gained considerable attention in recent years within social and political psychology due to their influence on a wide range of attitudes, behaviors, and intergroup relations (Brotherton et al., 2013). From this perspective, several authors have argued that conspiracy beliefs are not merely isolated explanations for specific events but rather reflect a general psychological tendency to perceive social events as the result of secret actions orchestrated by powerful groups, a phenomenon commonly referred to as conspiracy mentality (Bruder et al., 2013).

Although conspiracy beliefs have traditionally been associated with governments, powerful economic actors, public health crises, or global political events, recent research has highlighted the emergence of conspiracy narratives targeting specific social groups (Douglas et al., 2019; Jolley et al., 2020). These beliefs are particularly relevant because they contribute to the justification and maintenance of prejudice, discrimination, and social exclusion (Douglas et al., 2017). Several studies have shown that exposure to conspiracy theories concerning specific social groups increases intergroup prejudice, promotes social exclusion, and may even generalize negative attitudes toward other minority groups not directly involved in the conspiracy narrative (Jolley et al., 2020).

This interpretation is consistent with the assumptions of Intergroup Threat Theory, which proposes that prejudice toward minority groups increases when they are perceived as threatening the values, resources, or social organization of the majority group (Stephan & Stephan, 2000). From this perspective, conspiracy narratives about LGBTQ+ individuals can be understood as expressions of symbolic threat associated with perceived challenges to cultural norms, traditional family structures, and established value systems.

Among the social groups targeted by these contemporary forms of conspiracy beliefs are lesbian, gay, bisexual, transgender, and queer (LGBTQ+) individuals (Panerati & Salvati, 2025; Salvati et al., 2024a, 2024b). Conspiracy beliefs directed toward LGBTQ+ communities portray them as organized groups seeking disproportionate influence over political institutions, educational systems, media outlets, and legal frameworks (Salvati et al., 2024b).

In public discourse, these conspiracy beliefs are frequently expressed through narratives attributing deliberate social change to a supposed “gender ideology,” denouncing processes of ideological indoctrination and claiming that LGBTQ+ organizations pursue hidden political agendas aimed at transforming traditional norms and values (Salvati et al., 2024b; Valsecchi et al., 2025).

These beliefs can be understood as a contemporary manifestation of intergroup prejudice. Similar to other conspiracy theories directed against political or social groups, they attribute secret coordination, hidden intentions, and excessive power to a socially identifiable outgroup (Brotherton et al., 2013). Recent experimental evidence further supports the intergroup relevance of these beliefs, showing that exposure to anti-LGBTQ+ conspiratorial narratives can increase their endorsement and that such beliefs are associated with lower support for inclusive initiatives and reduced solidarity with LGBTQ+ individuals (Panerati et al., 2026). This interpretation is consistent with the literature on contemporary forms of prejudice toward sexual minorities. Unlike traditional expressions of overt rejection, modern forms of prejudice often manifest through narratives that question the legitimacy of equality claims, exaggerate the political influence of LGBTQ+ groups, or portray these communities as receiving unfair advantages within society (Bettinsoli et al., 2022; Morrison & Morrison, 2003).

Previous research suggests that conspiracy beliefs may be embedded in broader ideological processes related to the maintenance of existing social hierarchies and resistance to social change. In line with this view, conspiracy beliefs have been associated with ideological orientations such as right-wing authoritarianism, social dominance orientation, and generalized prejudice toward minority groups (De Cristofaro et al., 2025; Salvati et al., 2024a).

Despite the increasing visibility of conspiracy beliefs targeting LGBTQ+ communities, empirical evidence remains limited due to the lack of instruments specifically designed to assess such beliefs. Previous research has often relied on broad indicators, such as beliefs concerning a supposed “gay agenda,” which are insufficient for capturing the complexity of conspiracy beliefs directed toward LGBTQ+ groups (Bettinsoli et al., 2022).

To address this limitation, Salvati et al. (2024b) developed the Gender Ideology and LGBTQ+ Lobby Conspiracies Scale (GILC). The GILC is a brief self-report measure designed to assess beliefs regarding alleged conspiratorial actions carried out by LGBTQ+ individuals and organizations. The instrument consists of nine items assessing perceptions of hidden influence, political manipulation, ideological imposition, and coordinated action attributed to LGBTQ+ groups. In the original study, Salvati et al. (2024b) reported a unidimensional structure with excellent psychometric properties, high internal consistency, and measurement invariance across sex assigned at birth and sexual orientation.

Subsequent studies have employed the instrument without structural modifications and have reported findings consistent with the original validation, including excellent reliability indicators across different samples (De Cristofaro et al., 2025; Panerati & Salvati, 2025; Salvati et al., 2024a; Valsecchi et al., 2025). Collectively, this body of evidence suggests that conspiracy beliefs directed toward LGBTQ+ groups constitute a meaningful and distinct construct with important implications for intergroup relations.

Furthermore, these studies have shown that LGBTQ+-related conspiracy beliefs are associated with ideological positions emphasizing traditional norms and the defense of social hierarchies (Salvati et al., 2024a), lower support for LGBTQ+ equality (De Cristofaro et al., 2025), stronger conformity to traditional gender norms (Valsecchi et al., 2025), and broader patterns of negative sociopolitical attitudes (Salvati et al., 2024a). Conversely, positive intergroup contact and empathy toward LGBTQ+ individuals appear to reduce endorsement of conspiracy beliefs directed against these groups (Panerati & Salvati, 2025).

As noted above, despite the relevance of conspiracy beliefs for understanding intergroup relations and attitudes toward LGBTQ+ communities, empirical research in this area remains limited. In a French-speaking sample from Cameroon, Messanga et al. (2025) replicated the unidimensional structure of the scale but excluded two of the nine original items. These items explicitly referred to “gender ideology,” a concept that appeared to have limited cultural relevance within that context (Messanga et al., 2025). These findings highlight the importance of examining the generalizability of the measurement model across different sociocultural settings.

In summary, the limited number of validation studies represents an important obstacle to advancing research on conspiracy beliefs directed toward LGBTQ+ groups. This issue is particularly relevant in Spanish-speaking societies, where public debates concerning gender ideology, educational policies, and LGBTQ+ rights have become increasingly prominent.

Beyond addressing this measurement gap, the present study seeks to extend the theoretical understanding of LGBTQ+-related conspiracy beliefs to a Latin American sociocultural context. Most existing evidence has been generated in European samples, leaving unclear whether the ideological and intergroup processes associated with these beliefs generalize to contexts characterized by different political, religious, and cultural dynamics. Examining the GILC in Chile therefore provides an opportunity to assess whether LGBTQ+-related conspiracy beliefs are similarly embedded in broader ideological orientations and general conspiracist thinking. In this sense, the study contributes not only to the cross-cultural evaluation of the construct but also to understanding the extent to which anti-LGBTQ+ conspiracy beliefs represent a culturally transferable expression of contemporary intergroup prejudice.

The Chilean context is particularly relevant for examining these beliefs. In recent decades, Chile has experienced substantial changes in the social and legal recognition of sexual and gender diversity, while traditional gender role beliefs, religiosity, and negative attitudes toward sexual minorities remain relevant dimensions of social attitudes. Previous research in Chile has shown that stronger adherence to traditional gender role beliefs and religiosity is associated with more negative attitudes toward gay men and lesbians (Cárdenas et al., 2012). This coexistence of increasing recognition of LGBTQ+ rights and persistent traditional attitudes makes Chile a particularly informative context for examining the ideological and intergroup foundations of LGBTQ+-related conspiracy beliefs.

The present study aimed to adapt and validate the Spanish version of the Gender Ideology and LGBTQ+ Lobby Conspiracies Scale (GILC). Specifically, we examined evidence based on the internal structure of the instrument through parallel analysis, psychometric network analysis, confirmatory factor analysis, and measurement invariance testing across sex assigned at birth. In addition, we explored evidence based on relations with external variables, including age, educational attainment, occupational status, religiosity, and political orientation. Based on previous findings, we expected the scale to exhibit a unidimensional structure, demonstrate measurement invariance across sex assigned at birth, and show stronger endorsement among individuals reporting higher levels of religiosity and more right-wing political orientations (De Cristofaro et al., 2025; Salvati et al., 2024a). These sources of evidence are consistent with contemporary validity frameworks, which emphasize internal structure and relationships with external variables as fundamental components of construct validation (American Educational Research Association et al., 2014).

## 2. Materials and Methods

### 2.1. Participants

The sample comprised 611 Chilean adults recruited through convenience sampling via social media platforms and online networks in Antofagasta, Chile. Eligibility criteria included being at least 18 years of age and providing informed consent. No exclusion criteria were established beyond incomplete survey responses. Participants who did not complete the questionnaire were removed prior to analysis; therefore, no missing data were present in the final dataset.

Participants ranged in age from 18 to 78 years (M = 26.77, SD = 11.59). Sex assigned at birth was assessed for all participants. Of the total sample, 327 participants (53.5%) reported female sex assigned at birth, 265 (43.4%) reported male sex assigned at birth, and 19 (3.1%) selected “prefer not to answer”.

### 2.2. Instrument

#### 2.2.1. Sociodemographic and Ideological Variables

Sociodemographic information included age, sex assigned at birth, educational attainment, occupational status, frequency of religious participation, and political orientation. Sex assigned at birth was assessed using three response options: female, male, and prefer not to answer. These variables were included to examine evidence based on relations with external variables, given previous research linking conspiracy beliefs to demographic characteristics, religiosity, and ideological orientations (De Cristofaro et al., 2025; Salvati et al., 2024a). Age was recorded in years, whereas the remaining variables were assessed through self-report categorical measures.

#### 2.2.2. Gender Ideology and LGBTQ+ Lobby Conspiracies Scale (GILC)

The Gender Ideology and LGBTQ+ Lobby Conspiracies Scale (GILC; Salvati et al., 2024b) is a brief self-report measure designed to assess conspiracy beliefs directed toward LGBTQ+ individuals and organizations. The instrument consists of nine items rated on a five-point Likert scale ranging from 1 (strongly disagree) to 5 (strongly agree). Items assess perceptions of hidden influence, political manipulation, ideological indoctrination, and coordinated action attributed to LGBTQ+ groups.

In the original validation study, Salvati et al. (2024b) reported a unidimensional factor structure with high factor loadings, excellent internal consistency, and evidence of measurement invariance across sex assigned at birth and sexual orientation groups. These findings support the interpretation of the GILC as a reliable and structurally robust measure of LGBTQ+-related conspiracy beliefs.

The Spanish adaptation of the GILC followed the recommendations for test translation and adaptation proposed by Muñiz et al. (2013). The original items were translated into Spanish with particular attention to preserving their conceptual meaning and ensuring natural and comprehensible wording in the Chilean context. A back-translation procedure was subsequently conducted, and the back-translated version was reviewed by a bilingual translator with 12 years of professional experience, who assessed its correspondence with the original instrument and provided a written report on the adequacy of the translation. The comparison considered semantic, idiomatic, conceptual, and cultural equivalence. Subsequently, the complete questionnaire, including the adapted GILC, was pilot-tested with 32 participants to assess item comprehensibility and the practical administration of the questionnaire. No difficulties in item comprehension were identified during the pilot administration, and no modifications to the content or response format of the GILC were required before the main data collection.

#### 2.2.3. Generic Conspiracist Beliefs Scale (GCBS)

To examine convergent validity, participants completed the Spanish adaptation of the Generic Conspiracist Beliefs Scale (GCBS; Brotherton et al., 2013; López de Pomar & Lira Luttges, 2022). The GCBS comprises 15 items organized into five dimensions: government malfeasance, extraterrestrial cover-up, malevolent global conspiracies, personal well-being threats, and control of information. Responses are provided on a five-point Likert scale, with higher scores indicating stronger endorsement of conspiracy beliefs. Previous studies have reported satisfactory psychometric properties and high levels of internal consistency for the scale (Brotherton et al., 2013; López de Pomar & Lira Luttges, 2022).

### 2.3. Procedure

Participants were recruited through convenience sampling using online social networks and messaging platforms, specifically Instagram, Facebook, and WhatsApp. Data were collected during November and December 2025. The invitation included a link to the online questionnaire and briefly described the purpose of the study and eligibility requirements. Eligibility criteria included being at least 18 years old and providing informed consent. No additional exclusion criteria were applied, although only fully completed questionnaires were retained for analysis. Before accessing the questionnaire, participants were informed about the purpose of the study, the voluntary nature of participation, the confidentiality and anonymity of their responses, and their right to withdraw at any time without consequences. All participants provided electronic informed consent before beginning the survey. The study was approved by the Ethics Committee of Universidad Católica del Norte (Approval No. 081/2025) and was conducted in accordance with the ethical principles established in the Declaration of Helsinki.

### 2.4. Data Analysis

Statistical analyses were conducted using R version 4.5 and Mplus version 7. Polychoric correlation matrices were computed prior to the analyses due to the ordinal nature of the response scale. Evidence based on internal structure was examined through parallel analysis and psychometric network analysis using the GLASSO estimator in R. Confirmatory factor analyses (CFA) and measurement invariance testing were performed in Mplus 7 using the robust weighted least squares estimator (WLSMV). Model fit was evaluated using the Comparative Fit Index (CFI), Tucker–Lewis Index (TLI), and Root Mean Square Error of Approximation (RMSEA), following commonly accepted guidelines. Composite reliability (CR) and average variance extracted (AVE) were estimated to evaluate internal consistency and convergent validity following the recommendations of Fornell and Larcker (1981). Measurement invariance across sex assigned at birth was assessed sequentially through configural, metric, and scalar models. Evidence based on relations with external variables was examined using Pearson correlations, independent-samples *t*-tests, one-way analyses of variance (ANOVA), Welch’s robust tests when homogeneity of variance assumptions were violated, and Games–Howell post hoc comparisons. Statistical significance was set at *p* < 0.05.

## 3. Results

### 3.1. Evidence Based on Internal Structure

**Figure 1:**
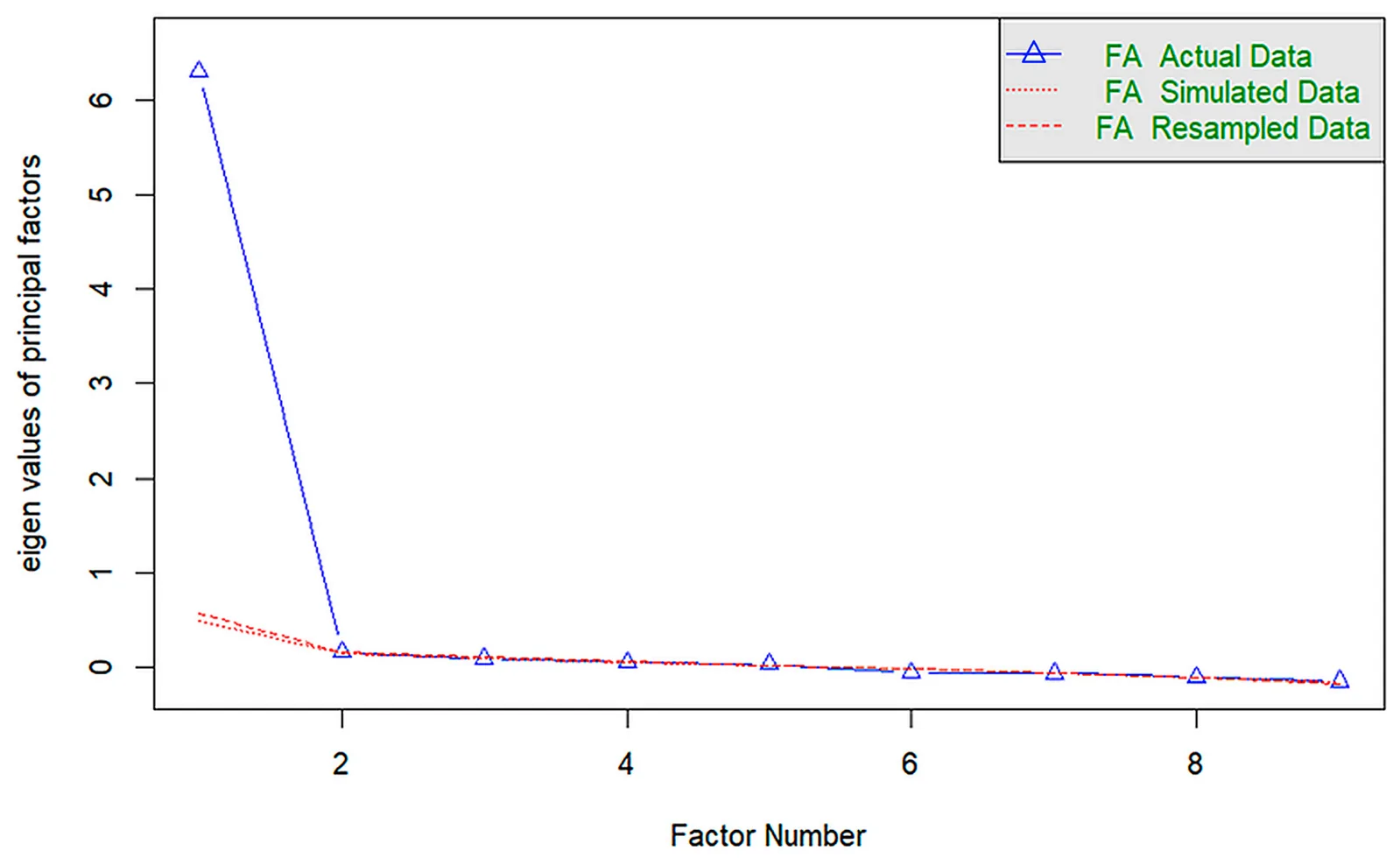
Parallel analysis.

**Figure 2:**
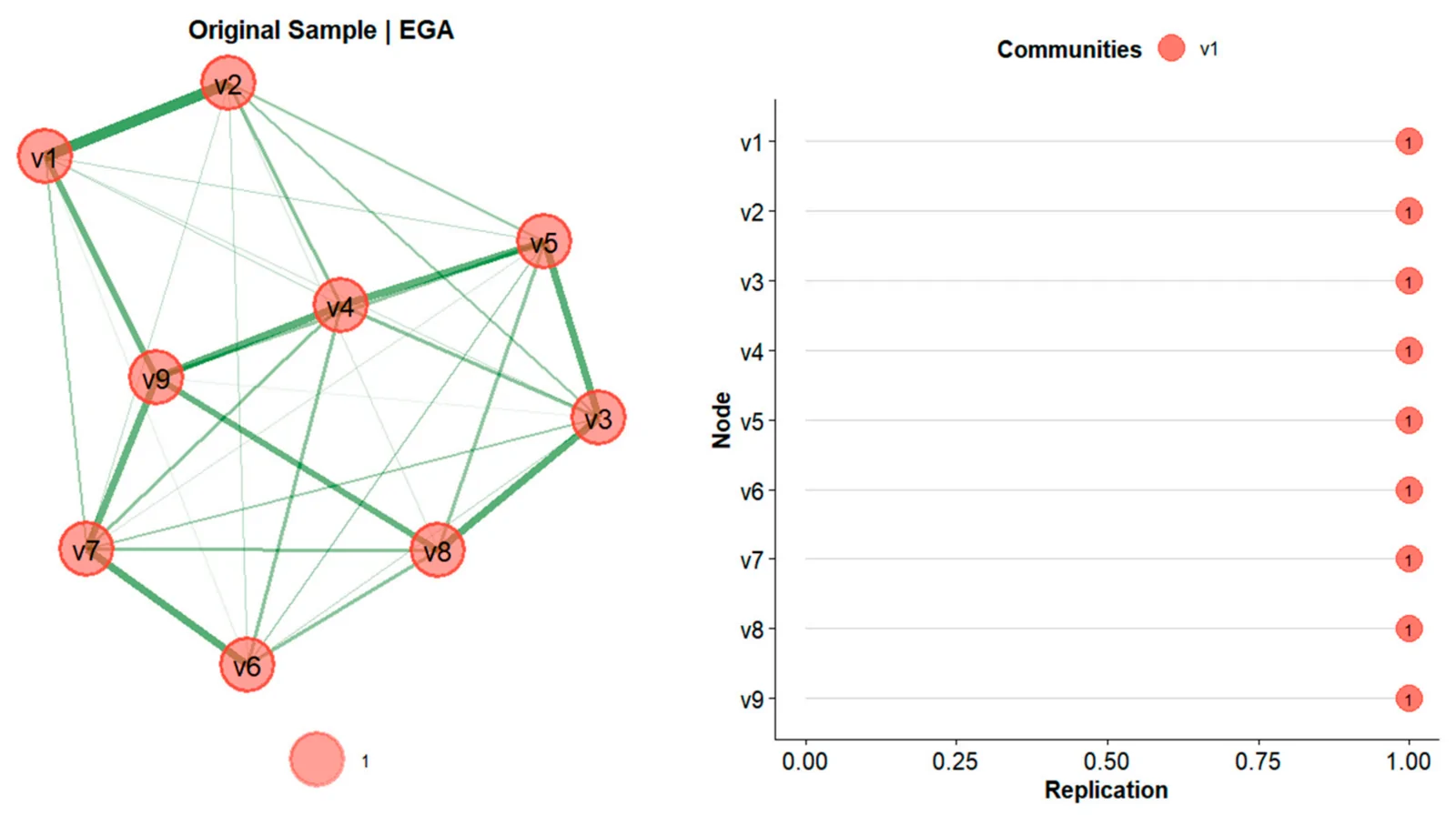
Psychometric network estimated using GLASSO.

The internal structure of the GILC was examined using complementary analytical approaches. First, a parallel analysis based on minimum residual extraction supported the retention of a single factor, as only the first empirical eigenvalue exceeded the corresponding random eigenvalue (Figure 1). Consistent with this finding, psychometric network analysis estimated using the GLASSO algorithm identified a single community of nodes with 100% stability across bootstrap replications (Figure 2), supporting the existence of a dominant latent dimension.

To further evaluate the dimensionality of the instrument, a series of confirmatory factor analyses were estimated using WLSMV. The original one-factor model showed acceptable incremental fit indices but an elevated RMSEA. Two theoretically interpretable residual correlations were subsequently incorporated, resulting in significant improvements in model fit. The final model showed the best overall fit and was retained for subsequent analyses (Table 1).

**Figure 3:**
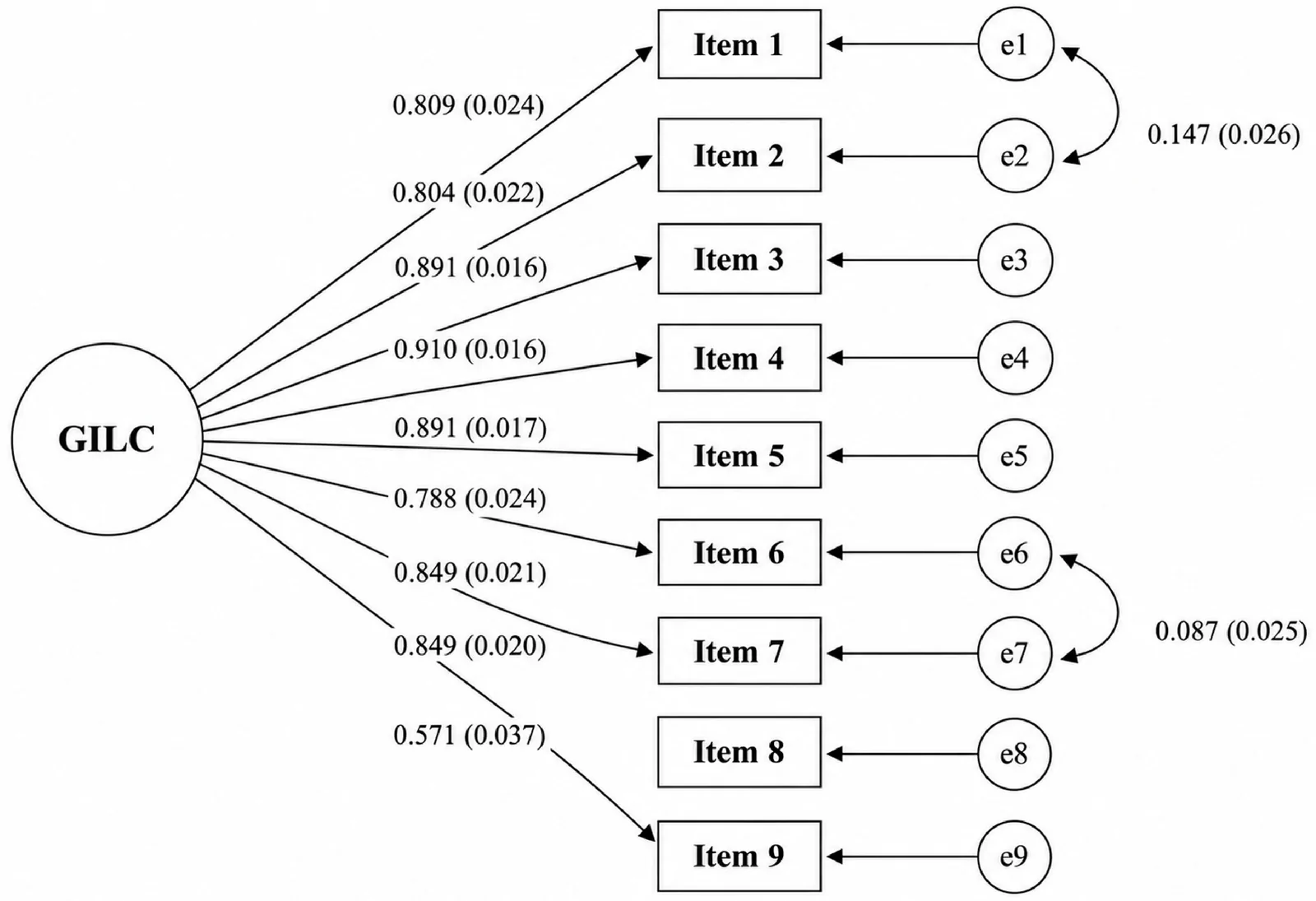
Standardized CFA solution of the final model.

The standardized solution of the final model is presented in Figure 3 and Table 2. Although two residual correlations were required, the convergence of exploratory, network, and confirmatory approaches supports the interpretation of the GILC as an essentially unidimensional measure of LGBTQ+-related conspiracy beliefs.

The residual correlation between Items 1 and 2 suggests the presence of shared variance associated with the attribution of political influence and strategic power to organized LGBTQ+ actors. Likewise, the residual correlation between Items 6 and 7 appears to reflect a common content related to the perceived use of institutions and legal mechanisms to impose ideological agendas. These residual associations likely reflect semantic overlap and item content similarity rather than the existence of substantively distinct dimensions. These residual correlations are more parsimoniously interpreted as local item dependence arising from semantic overlap rather than evidence of additional latent dimensions. Given the convergence of the parallel analysis, network analysis, and confirmatory factor analysis, the findings support the interpretation of the GILC as an essentially unidimensional measure of LGBTQ+-related conspiracy beliefs. Internal consistency estimates indicated excellent reliability and convergent validity for the scale (McDonald’s ω = 0.97, Cronbach’s α = 0.95, AVE = 0.75, and CR = 0.97). These findings suggest that the items consistently reflect a common underlying construct.

The correlated residuals were theoretically supported by the semantic overlap between the corresponding item pairs. Items 1 and 2 both refer to the alleged acquisition and exercise of political influence by LGBTQ+ individuals or groups, whereas Items 6 and 7 both describe the alleged imposition of a particular ideological or political worldview. Thus, these residual associations may reflect localized shared content beyond the general conspiracy-belief factor rather than evidence of additional latent dimensions.

Measurement invariance across sex assigned at birth was then evaluated. Results supported configural, metric, and scalar invariance, indicating that the scale operates equivalently across men and women (Table 3).

Finally, men (M = 2.69, SD = 1.09) obtained significantly higher GILC scores than women (2.32, SD = 1.01), t_(590)_ = 4.219, *p* < 0.001, although the magnitude of the difference was small to moderate (d = 0.35, 95%CI [0.19, 0.51]). This finding is consistent with previous research showing that men tend to express more negative attitudes toward sexual minorities than women (Kite & Whitley, 1996). The presence of scalar invariance indicates that this difference is unlikely to reflect measurement bias and instead reflects meaningful group differences in the endorsement of LGBTQ+-related conspiracy beliefs.

### 3.2. Relations with External Variables

Evidence based on relations with external variables was examined through correlations and group comparisons (Table 4). GILC scores were positively associated with generic conspiracy beliefs, *r* = 0.435, *p* < 0.001, providing support for convergent validity.

Age showed a small but significant positive association with GILC scores, *r* = 0.209, *p* < 0.001. In contrast, no significant differences were observed according to educational attainment, Welch’s *F*_(6, 58.08)_ = 0.87, *p* = 0.523, or occupational status, Welch’s *F*_(5, 100.70)_ = 0.94, *p* = 0.459.

**Figure 4:**
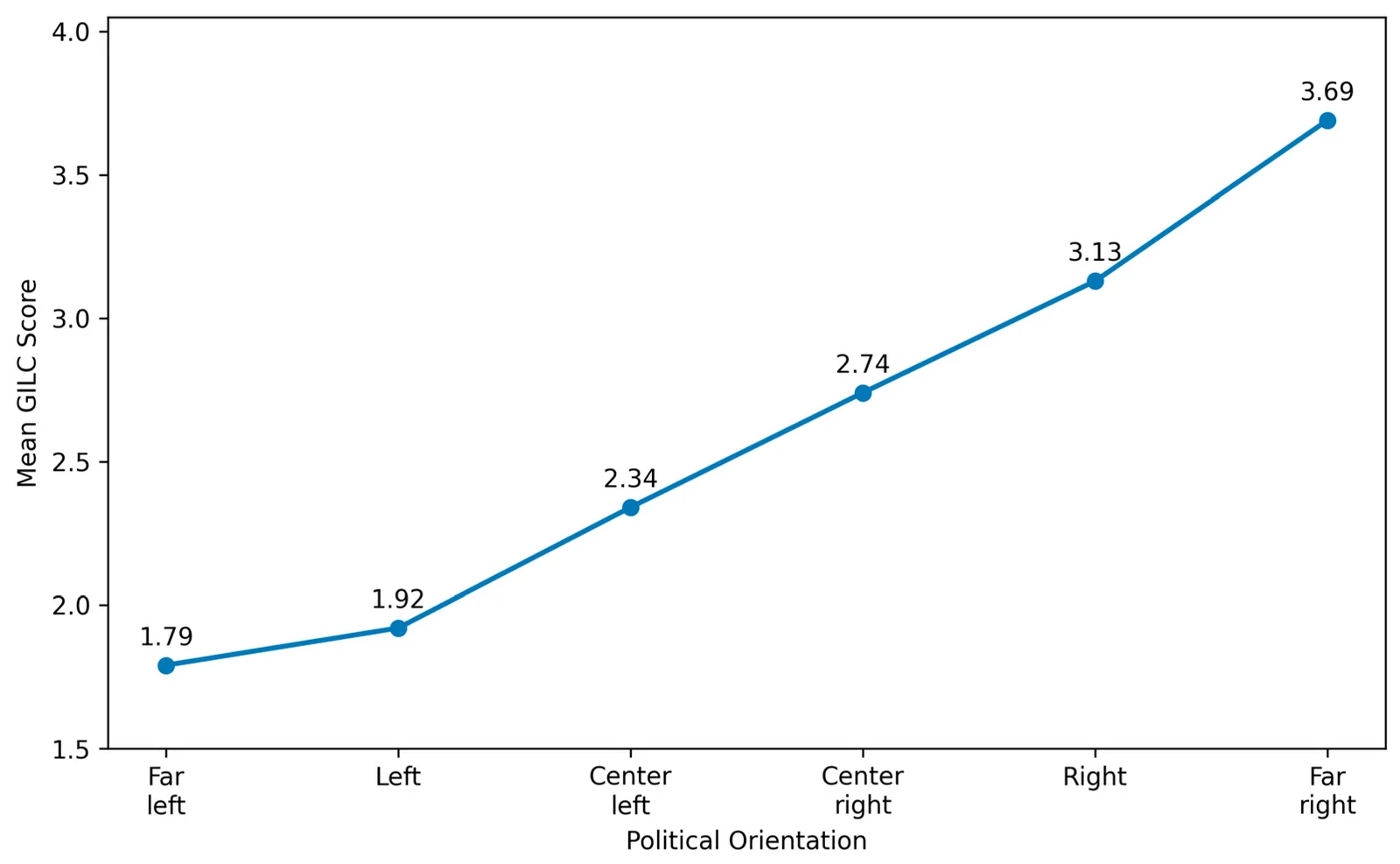
Mean GILC scores according to political orientation.

Significant differences emerged for both religious participation and political orientation. Individuals reporting more frequent participation in religious activities tended to endorse higher levels of LGBTQ+-related conspiracy beliefs, Welch’s *F*_(6, 51.58)_ = 7.32, *p* < 0.001, η^2^ = 0.07. Likewise, political orientation showed the strongest association with GILC scores, Welch’s *F*_(5, 104.00)_ = 27.45, *p* < 0.001, η^2^ = 0.18, with mean scores increasing progressively from the extreme-left to the extreme-right categories (Figure 4).

Taken together, these associations provide additional validity evidence based on relations with theoretically relevant external variables. In particular, the association with general conspiracy beliefs supports the expected convergence between LGBTQ+-related conspiracy beliefs and a broader conspiracist worldview, whereas the associations with political orientation and religious participation are consistent with the proposed ideological correlates of the construct.

## 4. Discussion

The present study aimed to adapt and evaluate the validity of the Spanish version of the Gender Ideology and LGBTQ + Lobby Conspiracies Scale (GILC) in a Chilean sample. Overall, the findings provide favorable evidence supporting the use of the instrument in Spanish-speaking contexts. The analyses supported a predominantly unidimensional structure, demonstrated adequate measurement equivalence across men and women, and revealed meaningful relationships with theoretically relevant external variables. These results are consistent with previous findings reported in other cultural contexts (De Cristofaro et al., 2025; Salvati et al., 2024a, 2024b). Taken together, these findings provide favorable validity evidence based on internal structure and relationships with external variables, two of the principal sources of validity evidence identified in contemporary testing standards (American Educational Research Association et al., 2014).

Evidence based on internal structure showed a high degree of convergence across different analytical approaches. Both parallel analysis and psychometric network analysis supported the presence of a single dominant dimension, a finding that was subsequently confirmed through confirmatory factor analysis. Although the final model yielded an RMSEA slightly above conventional cutoffs, the excellent CFI and TLI values, together with the convergence of the parallel analysis and psychometric network analysis, support the adequacy of the proposed unidimensional structure. The two residual correlations incorporated into the final model involved item pairs with conceptually similar content. Specifically, Items 1 and 2 both refer to the alleged acquisition and exercise of political influence by LGBTQ+ individuals or groups, whereas Items 6 and 7 both describe the alleged imposition of a particular ideological or political worldview. These localized residual associations can therefore be interpreted as reflecting shared semantic content beyond the general conspiracy-belief factor rather than substantively distinct latent dimensions. Importantly, the residual correlations were relatively small and did not contradict the broader evidence of unidimensionality provided by the parallel analysis and psychometric network analysis.

A particularly relevant finding was the evidence of configural, metric, and scalar invariance across sex assigned at birth. From a psychometric perspective, this indicates that the scale functions equivalently for men and women, allowing meaningful comparisons of observed and latent scores across groups. This finding is consistent with evidence reported for the original version of the instrument and suggests that its measurement properties remain stable across different cultural settings.

Regarding relationships with external variables, the positive association observed between the GILC and the Generic Conspiracist Beliefs Scale provides favorable evidence of convergent validity. The moderate magnitude of this relationship suggests that both measures share a common conceptual core related to a general tendency to endorse conspiratorial explanations, while remaining empirically distinct. This finding is consistent with theoretical perspectives that distinguish between a broad conspiratorial mindset and specific manifestations of conspiracy beliefs directed toward particular social groups or issues.

The analyses also revealed significant associations between LGBTQ+-related conspiracy beliefs and several sociodemographic and ideological variables. Specifically, higher levels of endorsement were associated with older age, greater religious participation, and more right-wing political orientation. These findings are consistent with previous research linking LGBTQ+-related conspiracy beliefs to more conservative ideological positions and stronger adherence to traditional values (De Cristofaro et al., 2025; Salvati et al., 2024a). They are also consistent with evidence from the Chilean context showing that religiosity and traditional gender role beliefs are associated with more negative attitudes toward sexual minorities (Cárdenas et al., 2012). This interpretation is further supported by research showing that the relationship between religiosity and opposition to LGBTQ+ rights is intertwined with sexual prejudice and conservative ideological orientations, particularly resistance to social change (van der Toorn et al., 2017). Similar associations between conservatism, religiosity, beliefs about sexual orientation, and sexual prejudice have also been observed in a Brazilian sample, providing further evidence of these relationships within a Latin American context (Santos et al., 2026). Among the variables examined, political orientation showed the strongest association with GILC scores. Notably, this pattern contrasted with the absence of significant differences according to educational attainment and occupational status. Taken together, these findings suggest that endorsement of LGBTQ+-related conspiracy beliefs may be more closely related to ideological positioning and value-laden worldviews than to structural sociodemographic characteristics. In this sense, these beliefs may reflect broader sociopolitical orientations concerning gender, sexuality, and social change rather than simply differences in educational or occupational background. The magnitude of the association with political orientation further suggests that ideological positioning may constitute one of the most important correlates of LGBTQ+-related conspiracy beliefs.

A significant difference was observed between men and women, with men reporting slightly higher levels of LGBTQ+-related conspiracy beliefs. Although the effect size was modest, this finding is consistent with previous research indicating that men tend to express more negative attitudes toward sexual minorities and gender-diverse groups than women (Kite & Whitley, 1996). Because scalar invariance was supported, this difference is unlikely to reflect measurement bias. Nevertheless, the present study did not include variables that could clarify the mechanisms underlying this association, such as sexual orientation, attitudes toward gender equality, or endorsement of traditional gender norms. Future research should examine whether these factors help explain sex differences in LGBTQ+-related conspiracy beliefs.

From a cross-cultural perspective, the pattern observed in Chile appears broadly consistent with findings reported in previous studies conducted in European contexts, where LGBTQ+-related conspiracy beliefs have also been associated with conservative ideological orientations and religiosity. This convergence is noteworthy given the different sociopolitical and cultural contexts in which these studies were conducted and suggests that some of the ideological correlates of LGBTQ+-related conspiracy beliefs may extend beyond the specific national setting in which the construct was originally examined. At the same time, the Chilean context may give particular meaning to these associations. The coexistence of substantial advances in the recognition of sexual and gender diversity with the persistence of traditional religious and gender role beliefs may provide a sociocultural environment in which narratives concerning “gender ideology” and LGBTQ+ political influence acquire particular salience. Thus, the present findings suggest both cross-cultural continuity in the ideological correlates of LGBTQ+-related conspiracy beliefs and the importance of considering the specific sociopolitical context in which these beliefs are expressed.

Several limitations should be acknowledged. First, the sample was obtained through non-probability convenience sampling using online recruitment and consisted predominantly of relatively young adults. Recruitment through social media and messaging platforms may have favored the participation of individuals with greater access to and familiarity with digital technologies and may have introduced self-selection bias, as participation depended on individuals voluntarily responding to an invitation concerning the study topic. Consequently, the sample should not be considered representative of the broader Chilean population, and caution is warranted when generalizing the present findings, particularly to older age groups. Future studies using probability-based or more demographically diverse sampling strategies are needed to examine the robustness and generalizability of these findings across the Chilean population. Second, the cross-sectional design precludes causal interpretations regarding the relationships among the variables examined. Third, all measures were based on self-report, which may have introduced response biases, particularly given the socially sensitive nature of attitudes and beliefs concerning LGBTQ+ issues. Participants may have underreported or modified their responses because of social desirability concerns. Moreover, the use of a common self-report method for assessing the principal study variables may have contributed to common method variance, potentially inflating some of the observed associations. Future research could address these limitations by incorporating measures of social desirability and using complementary assessment methods or multiple sources of information. Fourth, the study assessed sex assigned at birth but did not collect information on sexual orientation or gender identity. Given the focus of the GILC, this limits our ability to examine the psychometric functioning of the scale across sexual orientation and among gender-diverse populations. Future studies should therefore incorporate measures of sexual orientation and more inclusive measures of gender identity and examine measurement invariance across these groups. Fifth, although the overall evidence supported an essentially unidimensional structure, the final CFA model required two theoretically justified residual correlations to achieve an acceptable fit, and the RMSEA remained slightly above conventional cutoff values. These findings suggest that some caution is warranted when interpreting the factorial structure and highlight the need to replicate the measurement model in independent samples. Sixth, information on other relevant psychosocial variables was not collected, restricting a more comprehensive understanding of the factors associated with these beliefs. Seventh, although the present study provides validity evidence based on the internal structure of the GILC and its associations with theoretically relevant external variables, other sources of validity evidence were not examined. In particular, discriminant validity against conceptually distinct constructs and predictive validity with respect to relevant behavioral or attitudinal outcomes should be examined in future studies.

Future research should extend the psychometric evaluation of the GILC in several directions. Longitudinal studies are needed to examine the temporal stability of LGBTQ+-related conspiracy beliefs and to determine whether changes in these beliefs are associated with changes in relevant ideological, social, or political variables. Relatedly, test–retest studies should evaluate the temporal reliability of GILC scores across repeated assessments. Cross-cultural research should also examine the measurement properties of the Spanish version across other Spanish-speaking countries, particularly through measurement invariance analyses, to determine whether the scale captures the same construct across different sociocultural contexts. Finally, future studies should evaluate measurement invariance and validity across more diverse demographic groups, including sexual orientation, age, educational level, and socioeconomic background. Such research would provide a stronger basis for establishing the temporal stability, cross-cultural generalizability, and demographic applicability of the Spanish GILC.

In conclusion, the Spanish version of the Gender Ideology and LGBTQ+ Lobby Conspiracies Scale provides favorable evidence of validity based on its internal structure, measurement invariance across sex assigned at birth, and relationships with theoretically relevant external variables. The findings suggest that the instrument constitutes a useful tool for the assessment of conspiracy beliefs directed toward LGBTQ+ individuals and groups in Spanish-speaking contexts, providing a foundation for future research on contemporary prejudice, ideological polarization, and intergroup relations.

## Figures and Tables

**Table 1:** Comparison of nested CFA models.

	χ^2^	df	*p*-Value	Δ_χ2_	Δ_df_	Δ_*p*-value_	CFI	TLI	RMSEA
Model A	260.730	27	0.000				0.980	0.973	0.146
Model B	135.521	26	0.000	47.847	1	0.000	0.991	0.987	0.102
Model C	99.445	25	0.000	30.110	1	0.000	0.994	0.991	0.086

Note. Model A = original unidimensional model; Model B = unidimensional model including a residual correlation between Items 1 and 2; Model C = unidimensional model including residual correlations between Items 1 and 2 and between Items 6 and 7. Improvements in model fit were evaluated using the DIFFTEST procedure for WLSMV estimation. CFI = Comparative Fit Index; TLI = Tucker–Lewis Index; RMSEA = Root Mean Square Error of Approximation.

**Table 2:** Standardized Factor Loadings and 95% Confidence Intervals for the One-Factor GILC Model.

Items	Standardized Factor Loading (λ)	95% CI
1. An organized group of LGBT people works for more power, hiding behind the demand for more rights.	0.809	[0.762, 0.856]
2. There are very powerful LGBT people who manage to influence the decisions of the Parliament and the Government, to the detriment of other citizens.	0.804	[0.761, 0.847]
3. Some very powerful people want to spread “gender ideology” in schools to indoctrinate children.	0.891	[0.860, 0.922]
4. A group of LGBT people has organized to infiltrate all major sectors of society to increase their influence on it.	0.910	[0.879, 0.941]
5. There are people who have organized themselves to subvert the natural order of things through “gender ideology”.	0.891	[0.858, 0.924]
6. There is an organization of some very powerful people who take advantage of LGBT instances to establish a dictatorship of single thought.	0.788	[0.741, 0.835]
7. LGBT people want to use laws and courts to impose a precise political view of the world.	0.849	[0.808, 0.890]
8. There are concrete propaganda attempts in schools to plagiarize children and allow them to decide whether to be male or female as they wish.	0.849	[0.810, 0.888]
9. LGBT people want to enact laws to favour themselves economically, professionally, socially, to the detriment of heterosexual people.	0.571	[0.498, 0.644]

Note. λ = standardized factor loading; CI = confidence interval. Item wording corresponds to the original English version of the GILC; the Spanish adapted version administered in the present study is provided in the Appendix A.

**Table 3:** Measurement invariance across sex assigned at birth.

	χ^2^	df	*p*-Value	Δ_χ2_	Δ_df_	Δ_*p*-value_	CFI	TLI	RMSEA
Configural	140.050	67	0.000				0.993	0.993	0.075
Metric	136.178	76	0.000	16.974	9	0.049	0.994	0.995	0.064
Scalar	128.399	85	0.002	9.496	9	0.392	0.996	0.997	0.052

Note: Men (M = 2.69, SD = 1.09) scored significantly higher than women (M = 2.32, SD = 1.01), t_(590)_ = 4.219, *p* < 0.001, d = 0.35.

**Table 4:** Evidence Based on Relations Between GILC Scores and External Variables.

Variable	Statistic	*p*-Value	Effect Size	95% CI
Generic conspiracy beliefs	—	<0.001	*r* = 0.435	[0.368, 0.498]
Age	—	<0.001	*r* = 0.209	[0.132, 0.283]
Educational level	Welch *F*_(6, 58.08)_ = 0.87	0.523	η^2^ = 0.010	[0.005, 0.044]
Occupational status	Welch *F*_(5, 100.70)_ = 0.94	0.459	η^2^ = 0.008	[0.003, 0.038]
Religious participation	Welch *F*_(6, 51.58)_ = 7.32	<0.001	η^2^ = 0.070	[0.043, 0.121]
Political orientation	Welch *F*_(5, 104.00)_ = 27.45	<0.001	η^2^ = 0.184	[0.137, 0.247]

Note. CI = confidence interval. Confidence intervals for Pearson’s *r* were calculated using Fisher’s z transformation. Confidence intervals for η^2^ were estimated using nonparametric bootstrap resampling.

## Data Availability

Due to ethical and legal restrictions related to participant confidentiality and the conditions established in the informed consent procedure, the data underlying this study cannot be made publicly available. The Ethics Committee approval did not include authorization for public data sharing. The dataset may be made available by the corresponding author upon reasonable request and subject to approval by the Ethics Committee of Universidad Católica del Norte.

## Appendix A

**Table A1:** Spanish version of the Gender Ideology and LGBTQ+ Lobby Conspiracies Scale (GILC).

1. Un grupo organizado de personas LGBT trabaja para obtener más poder, ocultándose tras la demanda de más derechos.
2. Existen personas LGBT muy influyentes que logran incidir en decisiones del Congreso y del Gobierno, perjudicando a otros ciudadanos.
3. Algunas personas muy poderosas quieren difundir la ‘ideología de género’ en las escuelas para adoctrinar a los menores.
4. Un grupo de personas LGBT se ha organizado para infiltrarse en los principales ámbitos de la sociedad para aumentar su influencia.
5. Ciertas personas se han organizado para alterar el orden natural utilizando la “ideología de género”.
6. Hay una organización de personas muy poderosas que se aprovechan de las demandas LGBT para imponer una dictadura del pensamiento único.
7. Las personas LGBT buscan usar el sistema legal y los tribunales para imponer una visión política específica del mundo.
8. Existen intentos concretos de propaganda en las escuelas para manipular a los niños y permitirles decidir si quieren ser hombres o mujeres según lo deseen.
9. Las personas LGBT buscan aprobar leyes para beneficiarse económica, profesional y socialmente, en perjuicio de la gente heterosexual.
